# Characteristics and Sources of Heavy Metals in Tailings-Derived Surface Material in the Largest Lithium Mine in China by Reserves

**DOI:** 10.3390/jox16060188

**Published:** 2026-10-05

**Authors:** Yajun Xie, Yongqiang Liu, Zixuan Ye, Ning He

**Affiliations:** 1 Jiangxi Key Laboratory of Watershed Soil and Water Conservation, Jiangxi Provincial Academy of Water Resources Sciences, Nanchang 330029, China; 2 Field Observation and Research Station of Dongting Lake Natural Resource Ecosystem, Ministy of Natural Resources, Changsha 410007, China; 3 School of Civil and Environmental Engineering, Hunan University of Technology, Zhuzhou 412007, China; 4 Yichun Key Laboratory of Functional Agriculture and Ecological Environment, Yichun University, Yichun 336000, China

**Keywords:** lithium tailings, heavy metals, source apportionment, single-factor enrichment index, enrichment assessment

## Abstract

Lithium (Li) mining is one of the most critical strategic resources for sustainable development in the world. However, sources of heavy metals in Li mining have received limited attention. To explore the features and origins of heavy metals in Li mining tailings-derived surface material (TDSM), this study focused on the Yichun Li mine, China’s largest one by reserves. The heavy metal enrichment was evaluated by single-factor enrichment index, and the source apportionment of heavy metals was performed using the PMF (Positive Matrix Factorization) model. Mean concentrations were As 12.95 ± 1.94, Pb 55.79 ± 8.93, Cu 58.35 ± 5.42, Cd 20.16 ± 0.48, Zn 109.81 ± 3.67, Ni 44.25 ± 3.26, Co 21.01 ± 1.25, Mn 809.60 ± 32.33, Cr 88.29 ± 4.27, and V 101.97 ± 5.41 mg kg^−1^, respectively. Relative to the control samples, all heavy metals in TDSM exhibited varying degrees of enrichment. According to the single-factor enrichment index, the cadmium enrichment was the most severe (index = 130.55–215.55), while the enrichment levels of other heavy metals ranged from mild to moderate. Heavy metals were uniformly distributed within the 0–20 cm horizon, with no significant vertical variation. According to the PMF model, the potential sources of heavy metals were likely associated with mixed anthropogenic source, geogenic source associated with the weathering, and mining-related source, with estimated contribution rates of 25.7%, 43.8%, and 30.5% of the potential sources, respectively. These findings provide a theoretical basis for implementing source control measures for heavy metal enrichment in Li mining areas.

## 1. Introduction

Mineral development is the cornerstone of modern industrial society. However, the extraction and subsequent smelting of mineral resources can also lead to the environmental problem of heavy metal enrichment in mine soils [1,2]. Heavy metals in mine soils are persistent, non-biodegradable, and toxic pollutants [3,4]. Their accumulation can degrade soil quality, reduce microbial diversity, and impair plant growth and ecosystem stability [3,4]. Human exposure to these metals occurs mainly through food-chain transfer, dust inhalation, and dermal contact, while chronic exposure to Cd, Pb, and As can cause neurological, renal, cardiovascular, and carcinogenic effects [3,5]. Thus, the investigation and source apportionment of heavy metal contamination in mine soils has become a current international research hotspot [6].

Currently, studies on heavy metal enrichment in mine soils in China have covered various mine types, including coal, copper, antimony–arsenic, lead–zinc, manganese, gold, and iron mines [1,7,8,9,10,11,12,13]. Lithium (Li) is the most critical strategic resource in the sustainable development, therefore the environment protecting of Li mines during their development and utilization has special social significance [5,7]. Only a few studies have evaluated heavy metal contamination in Li mine soils, and they are conducted exclusively in certain regions. These studies confirm that Li mining and utilization also lead to varying degrees of soil heavy metal enrichment [6,7,8]. However, studies focused on specific areas, such as those in China [14,15], cannot represent the soil heavy metal pollution problems of the many Li mines in other regions, since the status of soil heavy metal enrichment may vary greatly between regions because of the differences in topography, climate, soil development, mining activities. Therefore, there is an urgent need to accumulate research cases from multiple regions.

Moreover, compared with studies on contamination assessment of heavy metals in Li mine soils, further source-oriented investigations of heavy metal enrichment in Li mine soils is rarely reported, and the sources of heavy metal enrichment in Li mine soils remain unclear [5,6,7].

China is the most important Li mining region in the world [5]. This study focused on the Yichun Li mine, China’s largest one by reserves [16]. During the processes of mining, beneficiation, and tailings disposal in this mine, heavy metals might be released into the surrounding environment. However, no previous study has systematically investigated the vertical distribution or source apportionment of heavy metals in the tailings-derived surface material (TDSM) [5]. To address this gap, this study integrates vertical distribution analysis, single-factor enrichment index, and Positive Matrix Factorization (PMF) model to study the heavy metal enrichment in TDSM. Rather than assessing contamination alone, this integrated approach links vertical distribution with quantitative source apportionment. The concentrations of 10 heavy metal elements (namely arsenic (As), lead (Pb), copper (Cu), cadmium (Cd), zinc (Zn), nickel (Ni), cobalt (Co), manganese (Mn), chromium (Cr), and vanadium (V)) in soils at different depths of the TDSM were determined. Although As is formally a metalloid, it is grouped with heavy metals in this study for consistency with common soil enrichment assessment practice. First, the vertical distribution characteristics of heavy metal element contents in TDSM were analyzed. Then, the single-factor enrichment index approach was applied to assess the degree of heavy metal enrichment in the TDSM. Finally, the PMF model was used to quantitatively analyze the potential sources of heavy metals and their contribution rates. The results are expected to provide source-oriented insights for identifying priority pollutants and quantifying their potential sources.

## 2. Materials and Methods

### 2.1. Sample Collection

Our study area, the Yichun Li mine (N 29°27′2.02″, E 112°47′32.28″), is located in Jiangxi Province, China. The Yichun Li mine has been exploited since the 1970s and remains in active operation, and the major minerals present are Li, tantalum (Ta), and niobium (Nb). In the valley below the mine, a tailings impoundment was established. The tailings pond occupies a valley-like terrain that is bounded by mountains on three sides and open to the northwest. Its current effective storage capacity is approximately 2 million cubic meters. Beyond Li, the ore contains significant amounts of associated elements, including Ta, Nb, rubidium (Rb), cesium (Cs), and heavy metals such as Cd, Pb, As, Cu, Mn, and Cr [17]. The tailings also retain a large proportion of the associated trace metals originally present in the ore, which can be mobilized under acidic or oxidative conditions and subsequently leach into surrounding soil and water systems [5,17].

**Figure 1:**
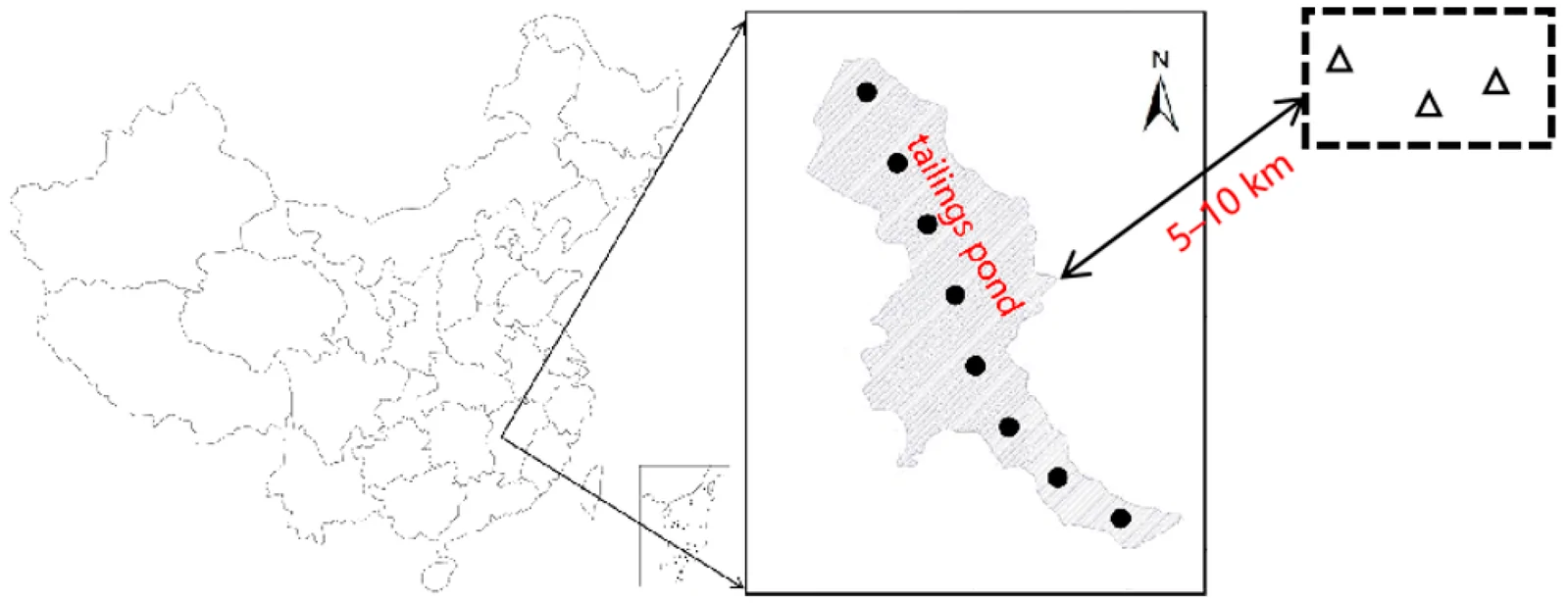
Study area, sampling points (solid black circles), and control sites (open triangles).

A total of 8 sampling points were selected from northwest to southeast in the Li tailings pond (Figure 1) [9]. The distance between two adjacent sampling points was approximately 100–200 m, and each sampling point was about 50–100 m from the edge of the tailings pond. The sampled material consists of weathered, hydraulically deposited flotation tailings rather than naturally developed soil. The tailings were discharged into the valley-type impoundment, where fine-grained particles settled and accumulated. Throughout this manuscript, this material is referred to as tailings-derived surface material (TDSM). The term “soil” is used only for the control sites and for general soil science concepts. At each sampling point, 5 quadrats were randomly selected, and one sample was collected from each quadrat. The sampling depth was 20 cm, and the samples were subdivided into 4 equal 5 cm depth intervals. For each of the four depth layers, TDSM cores from the five quadrats were thoroughly mixed to form one composite sample per depth layer per sampling point [18]. This yielded a total of 8 (points) × 4 (depths) = 32 composite TDSM samples for analysis.

The 8 sampling points were arranged along the major axis of the valley-shaped tailings pond (from northwest to southeast), which aligns with the main direction of tailings discharge and potential runoff flow [9]. Although this transect design cannot fully capture two-dimensional spatial heterogeneity, it is appropriate for detecting the primary gradient of heavy metal dispersion. Nevertheless, we acknowledge that a more extensive sampling grid (e.g., with additional points across the pond width) would improve spatial representativeness.

In order to further understand the current status of heavy metal contamination in the tailings, three control sites were selected from an undisturbed area located approximately 5–10 km from the mining area, beyond the influence of tailings discharge and prevailing wind dispersion. The control sites share the same parent material, topography, climate, and land-use history as the mining area, and show no visible signs of mining disturbance. The heavy metal concentrations at the control sites were close to the background values for Jiangxi Province, supporting their representativeness as local background references. Therefore, they are considered representative of the local geochemical background. The soil sampling method for the control samples was the same as that for the sampling points.

### 2.2. Analytical Methods

Samples were air-dried at room temperature (25 ± 2 °C) for 7 days, then ground in an agate mortar to pass through a 100-mesh nylon sieve [2,19]. All subsequent analyses were performed on the sieved fraction. A total of 10 heavy metal elements as mentioned above were measured, namely As, Pb, Cu, Cd, Zn, Ni, Co, Mn, Cr, and V. The Li concentrations in the TDSM ranged from 179 to 3302 mg kg^−1^ across study area. Li is not classified as a heavy metal, and its environmental mobility and ecotoxicity are generally lower than those of heavy metals [5]. Therefore, Li data are provided for reference only and are not used for enrichment assessment or PMF modelling. Nevertheless, Li can serve as a useful tracer of mining influence, and its inclusion in future risk assessments is recommended. For digestion, 0.5000 g of sample was treated with a 10 mL mixture of HNO_3_–HClO_4_–HF (10:5:5, *v*/*v*) on a hot plate (120 °C for 2 h, then 180 °C to near dryness), then the residue was dissolved in 2% HNO_3_ and diluted to 25 mL (HJ 832-2017 [20], the National Environmental Protection Standard of the People’s Republic of China). Concentrations of heavy metals were determined by ICP-AES (Thermo iCAP 6300, Waltham, MA, USA) [21,22]. The method offers a detection limit as low as 0.001 mg L^−1^. Calibration curves (0–10 mg L^−1^, r^2^ > 0.999) were prepared from commercial stock solutions diluted with 2% HNO_3_. A reagent blank was included every 20 samples. Duplicate analyses (10% of samples) gave Relative Percent Difference < 10%. Certified reference material (GBW07405, mine soil, Institute of Geophysical and Geochemical Exploration, Chinese Academy of Geological Sciences, Langfang, China) was analyzed in each batch (recoveries 92–108%, Table S1). The ring knife technique was used to determine water content and bulk density [22]. The pH was measured by a pH meter (model HQ411d, Hach Company, Loveland, CO, USA) with a 1:1 soil–water weight ratio [23]. Organic matter content was measured by the external heating method with H_2_SO_4_-K_2_Cr_2_O_7_ [10,22]. All concentrations are reported on a dry weight basis (105 °C, 24 h).

### 2.3. Heavy Metal Enrichment Assessment

The single-factor enrichment index method was applied to assess the degree of heavy metal enrichment [1,5]. The calculation formulas are as follows:

*P_i_* = *C_i_*/*S_i_* (1)

where *P_i_* denotes the single-factor enrichment index for element *i*, *C_i_* denotes the measured concentration of element *i* (mg kg^−1^), *S_i_* denotes the local background reference values for element *i* (mg kg^−1^), using the values of heavy metals in control sites. According to the numerical value of *P_i_*, heavy metal enrichment relative to control sites is classified into 5 grades, where *P_i_* ≤ 0.7 indicates below control sites, 0.7 < *P_i_* ≤ 1.0 indicates near control sites, 1.0 < *P_i_* ≤ 2.0 indicates mild enrichment, 2.0 < *P_i_* ≤ 3.0 indicates moderate enrichment, and *P_i_* > 3.0 indicates severe enrichment [11].

### 2.4. Statistical Analysis

Based on the results of Shapiro–Wilk tests for normality and Levene’s tests for homogeneity of variances (Table S2), variables that violated normality (organic matter, As, Cu, Pb, Cd) or homoscedasticity (Zn, Cd) were natural-logarithm transformed before further parametric analysis. Untransformed means and standard errors are presented in tables and figures for clarity.

A linear mixed-effects model was used with sampling site as a random intercept and depth as a fixed factor, since the four depth samples from the same site are not independent. Pairwise comparisons were performed using LSD. Then, correlations between TDSM properties were assessed using Pearson’s method. For correlation, site-level means (*n* = 8) were used. The above statistical analyses were performed using SPSS 22 (IBM Corporation, Armonk, NY, USA).

PMF was performed by EPA PMF 5.0 statistical software (U.S. Environmental Protection Agency, Washington, DC, USA; EPA PMF 5.0 User Guide). Uncertainty was calculated as $\sqrt{{\left(0.1\times concentration\right)}^{2}+{\left(detection\;limit/3\right)}^{2}}$. The model was run with 3 to 7 factors (sources), each with 20 random starts to avoid local minima. The final number of sources (*p* = 3) was selected based on: (i) the lowest QRobust/QTrue ratio; (ii) a sharp decrease in QRobust when increasing from 2 to 3 sources, followed by a plateau; (iii) physically interpretable factor profiles; (iv) residuals for most species within ±3. The 3-source solution also showed the highest bootstrap stability [24,25]. In model diagnostics, the QRobust (calculated excluding residuals > 4) and QTrue (using all residuals) were compared. For the 3-source solution, QRobust/QTrue was 0.92, indicating a good fit. Residuals for each metal were approximately normally distributed with mean near zero and 95% within ±3, confirming no systematic bias. Bootstrap (100 runs) was performed to evaluate source stability. Bootstrap factors were considered “mapped” if they showed >80% similarity to the base run factors. All three sources had mapping frequencies >95%. Displacement analysis was also conducted to check for local minima; no significant rotational ambiguity was detected.

## 3. Results and Discussion

### 3.1. Surface TDSM Physicochemical Properties

**Figure 2:**
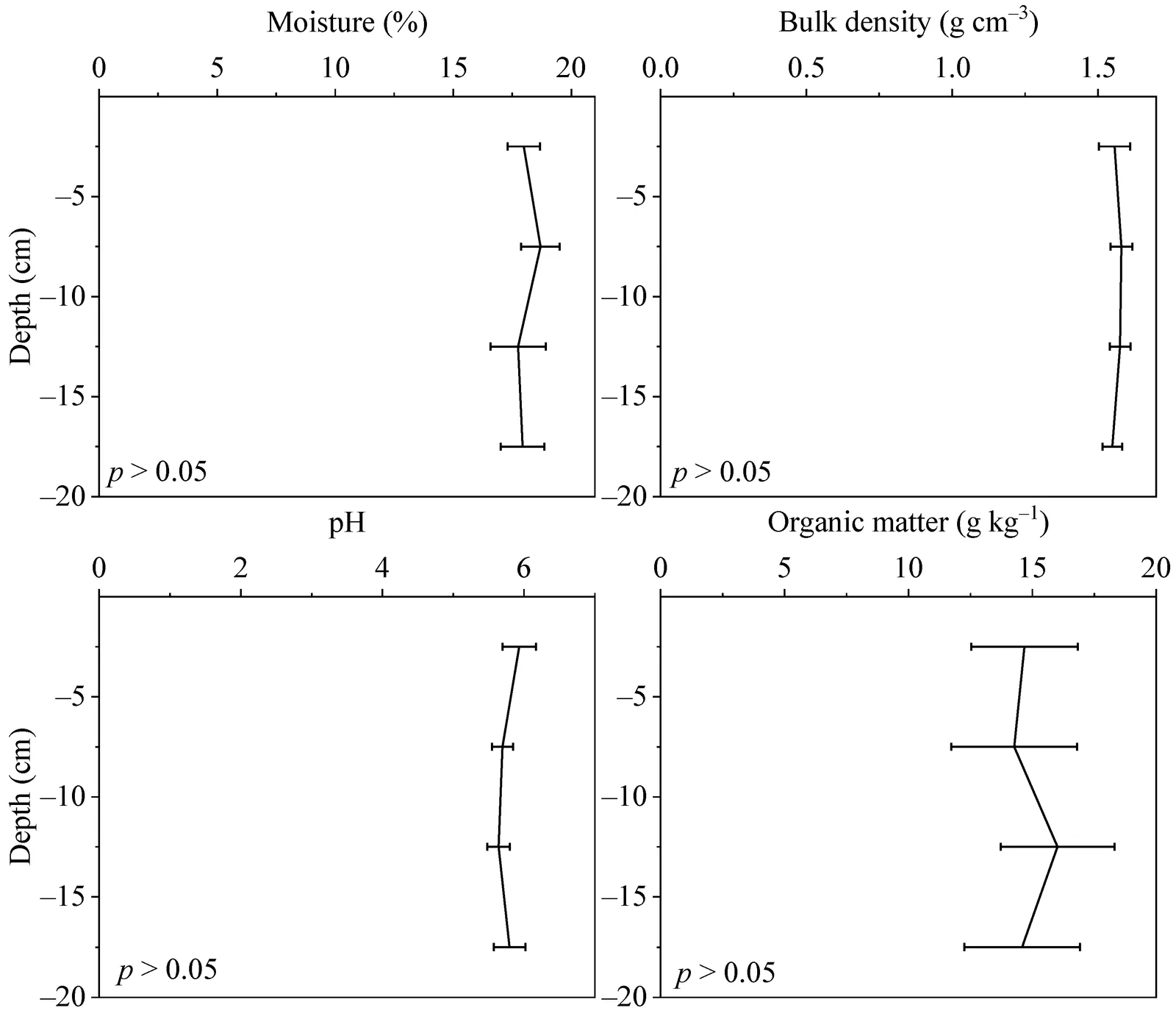
Vertical distribution of TDSM physicochemical characteristics (mean ± S.E.).

The TDSM of the Li tailings pond was generally weakly acidic, with an average pH of 5.76 ± 0.10. The mean values of moisture, bulk density, and organic matter content were 18.09 ± 0.44%, 1.57 ± 0.02 g cm^−3^, and 14.89 ± 1.12 g kg^−1^, respectively (Figure 2). Analysis of variance showed that these physicochemical properties had no significant differences with depth (*p* > 0.05, Figure 2).

### 3.2. Assessment of Surface TDSM Heavy Metal Enrichment

The average concentrations of TDSM As, Zn, Cu, Ni, Co, Mn, Cr, V, Pb, and Cd in the Yichun Li tailings pond were 12.95 ± 1.94 mg kg^−1^, 109.81 ± 3.67 mg kg^−1^, 58.35 ± 5.42 mg kg^−1^, 44.25 ± 3.26 mg kg^−1^, 21.01 ± 1.25 mg kg^−1^, 809.60 ± 32.33 mg kg^−1^, 88.29 ± 4.27 mg kg^−1^, 101.97 ± 5.41 mg kg^−1^, 55.79 ± 8.93 mg kg^−1^, and 20.16 ± 0.48 mg kg^−1^, respectively (Table 1). TDSM Pb and As had coefficients of variation of 90.57% and 84.54%, respectively, indicating high variability [26].

As suggested by the *P_i_*, As enrichment was the lowest among the 10 heavy metals in TDSM, with an average *P_i_*-As value of 1.11, and its enrichment level was classified as mild enrichment (Table 1). The concentrations of V, Zn, Co, Cr, and Pb in the TDSM were also at mild enrichment levels, while those of Ni, Mn, and Cu were at moderate enrichment levels. The concentration of Cd in the TDSM were under severe enrichment; the *P_i_*-Cd values fell in the range of 130.55 to 215.55, which were higher than the *P_i_* of each of the other heavy metals (Table 1). The concentrations of heavy metals were further compared with the risk control standard in China (GB15618-2018). The Cu content slightly exceeded the risk control standard, while the Cd content greatly exceeded it. The contents of As, Ni, Zn, Cr, and Pb did not exceed the standard.

Our findings demonstrate that heavy metal enrichment distinctly occurs in the surface TDSM of the Yichun Li tailings pond. Similar results have been observed elsewhere. For example, influenced by anthropogenic sources, heavy metals (e.g., Cd, As, Pb, Cr, Cu, Ni, Zn) have undergone some degree of enrichment in soils surrounding the beneficiation plant and tailings pond at Jiajika, Western Sichuan, China [7]. In this study, among the monitored heavy metal elements in the Yichun Li tailings pond, Cd had the most severe enrichment level relative to control sites. This is consistent with the conclusion that Cd currently stands as the dominant heavy metal contaminant across soils in China [27]. Studies on other types of mines have also supported the primary status of Cd among soil heavy metal pollutants [22,28,29].

### 3.3. Vertical Distribution of Surface TDSM Heavy Metals

**Figure 3:**
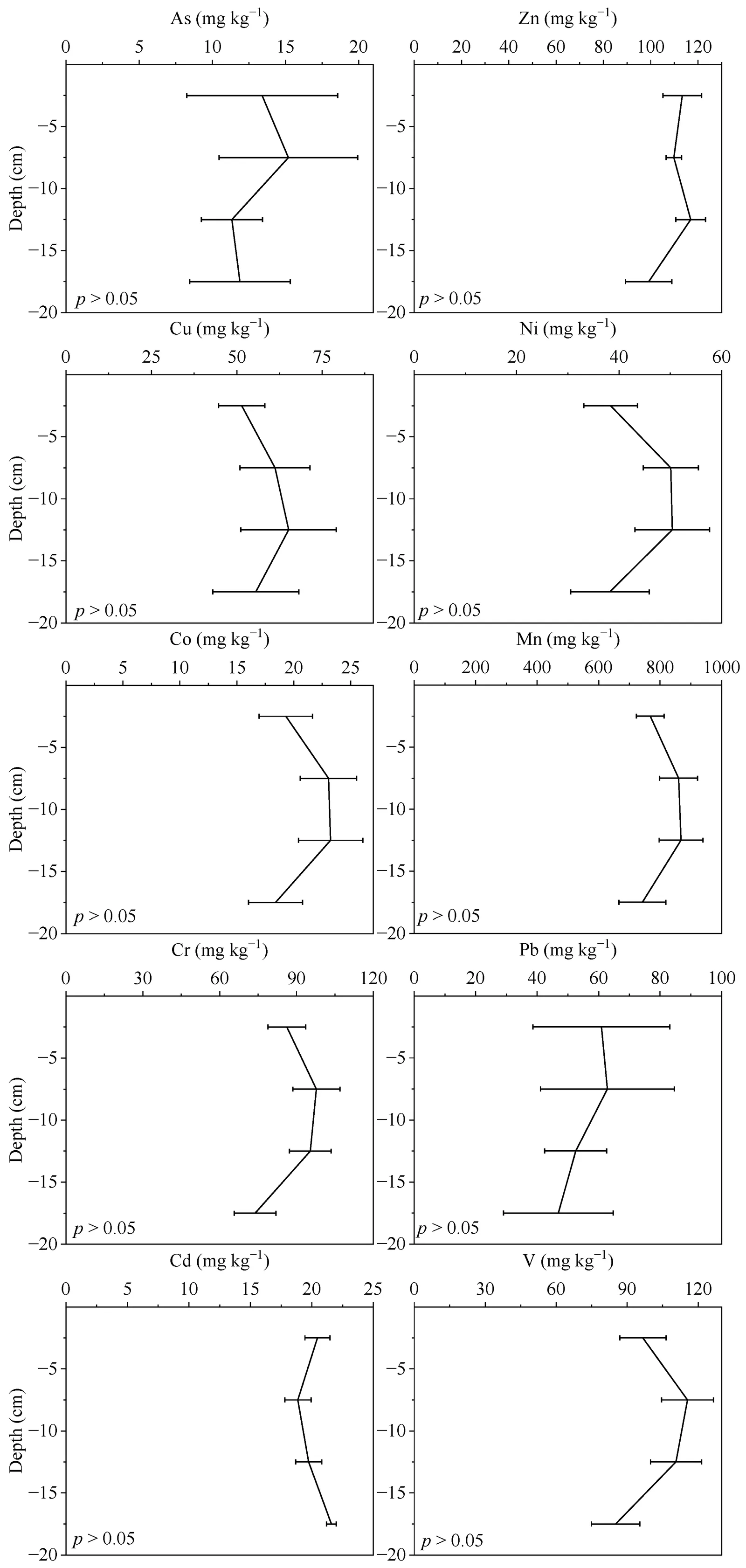
Depth profiles of TDSM heavy metal concentrations (mean ± S.E.).

Analysis of variance showed that the contents of measured heavy elements had no significant differences with TDSM depth (*p* > 0.05, Figure 3). This finding should be interpreted with caution and applies only to this shallow depth range. It does not imply uniform distribution throughout the entire TDSM profile. Some previous observations have studied the heavy metal distribution along the TDSM profile of the tailings pond. For example, in a closed gold mine tailings pond, the concentrations of heavy metals showed a decreasing trend from the top layer (0–10 cm) down to the 80–100 cm horizon [12], because heavy metal enrichment caused by human activities tends to be concentrated in the surface layer [12,30,31]. However, in the present study, the contents of the measured elements in the TDSM of the Li tailings pond showed no significant differences with depth. One reason might be the TDSM depth. The TDSM layers taken in this study were 0–20 cm, which is shallower than the depths selected in previous studies. Therefore, the heavy metal concentrations in the present study did not significantly decline with increasing TDSM depth. Another reason might be the origins of some heavy metals. As supported by the results of the potential source below, 43.8% of the heavy metals in this study may be emitted from natural sources. Compared with anthropogenic heavy metals, naturally sourced heavy metals (namely Ni, Co, V, Cr, and Cu) are more uniformly distributed with TDSM depth.

### 3.4. Relation Between TDSM Heavy Metal Concentrations and Physicochemical Characteristics

According to Pearson correlation analysis, there were positive or negative correlations between the concentrations of TDSM heavy metal elements in the Li tailings pond (Table 2). Among them, the concentrations of the other nine heavy metal elements either showed significant positive correlations (*p* < 0.05 or *p* < 0.01, e.g., between V and Ni, Co, Cr), or showed non-significant correlations (*p* > 0.05, e.g., between Pb and Cu, Ni, Co).

TDSM properties and heavy metal concentrations were also interrelated (Table 2). Bulk density was significantly negatively correlated with the concentrations of heavy metals including Co, Cr, and V (*p* < 0.05), suggesting that these elements are enriched in finer, less compacted fractions associated with clay minerals [30,31]. The pH exhibited a significant positive correlation with Cu and Mn (*p* < 0.05), likely because higher pH favors adsorption of these elements [32,33].

### 3.5. Potential Sources of TDSM Heavy Metals

**Figure 4:**
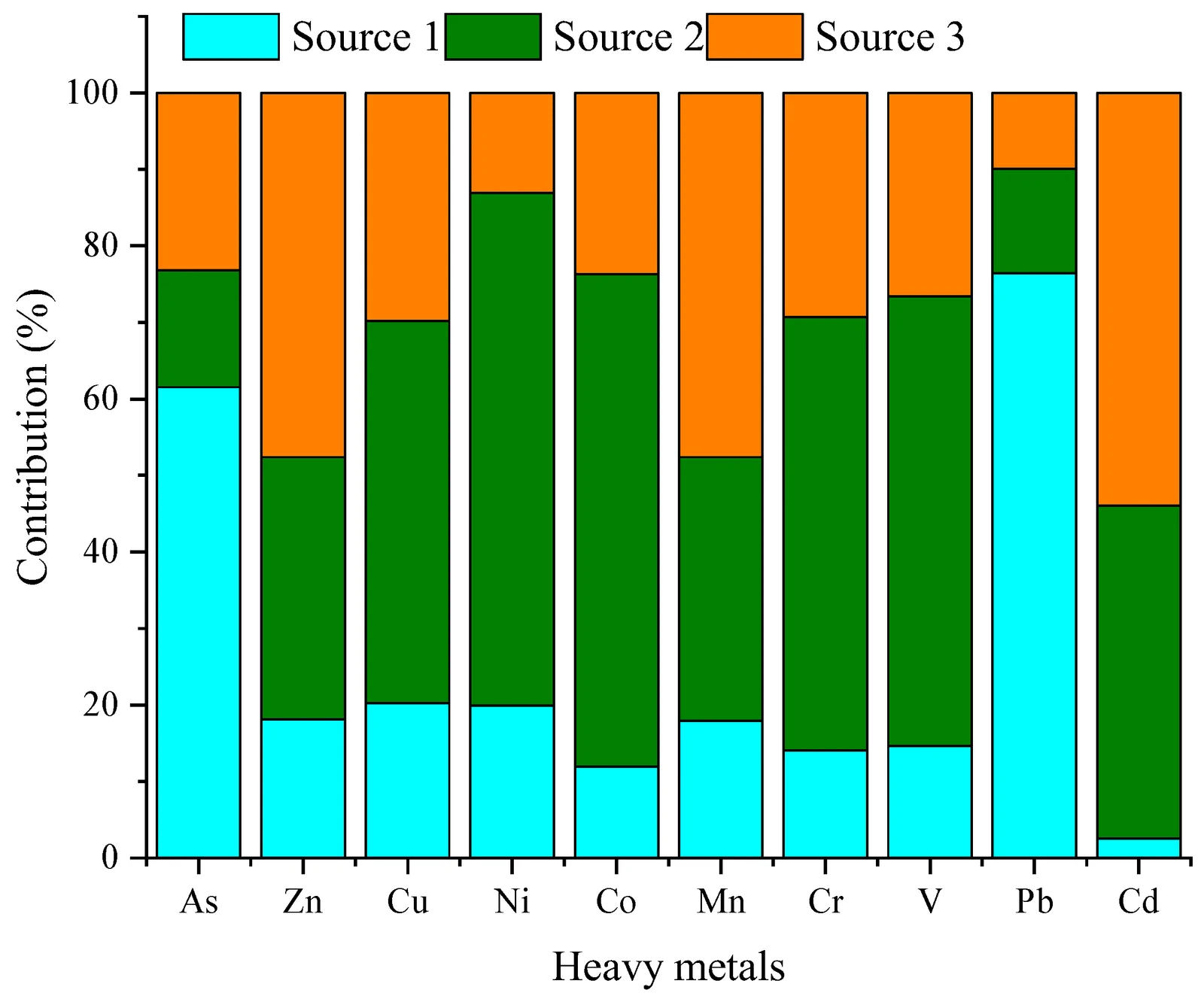
Contribution of TDSM heavy metals.

Since the heavy metals were uniformly distributed across the four TDSM layers with no significant vertical differences, when apportioning the potential sources of heavy metals, these four layers were treated as a single population, without considering differences in potential sources of heavy metals between different TDSM layers. The PMF was applied to statistically apportion the potential sources of heavy metals and their contribution rates (Figure 4), with the aid of other information of heavy metals such as enrichment levels, degrees of variation, potential sources list, and relationships with TDSM properties [24,25].

Among the three sources in the PMF, the Source 1 was estimated to account for 25.7% of the total potential source contribution of heavy metals. Source 1 was characterized by high loadings of Pb (76.4%) and As (61.51%), respectively (Figure 4). Among the ten measured heavy metals, Pb and As had the strongest spatial variability (Table 1), suggesting very uneven distribution. This suggests that these two heavy metals are greatly influenced by human activities and are unlikely to be from natural sources [26,34]. As suggested by the correlation analysis, a positive association existed between Pb and As (determination coefficient r^2^ = 0.958, Table 2), suggesting a common source for Pb and As [35]. In previous studies, the main potential anthropogenic sources of soil As are listed as arsenic-based pesticides, fertilizers, dust from fossil fuel combustion, and improper disposal of industrial and animal waste [32,33]; the main potential anthropogenic sources of soil Pb are lead-acid batteries and gasoline antiknock agents, followed by land application of sewage sludge and municipal waste, as well as mining and metal processing industries [36]. Given the presence of intense vehicle traffic and mining operations within the study area, Source 1 was tentatively interpreted as a mixed anthropogenic source related to combustion, traffic, and industrial dust, rather than being attributed solely to fossil fuel combustion. Previous studies have pointed out that Pb and As are typical volatile trace elements in fossil fuel combustion. During combustion, they volatilize, adsorb onto fine particulate matter (fly ash), are emitted into the atmosphere with flue gas, and then settle into soil or water bodies [33,36].

Source 2 explained 43.8% of the total source contribution and was characterized by high loadings of Ni (67.03%), Co (64.33%), V (58.83%), Cr (56.64%), and Cu (49.94%) (Figure 4). The Yichun deposit is a highly evolved Li–Ta-rich topaz–lepidolite granite [17], and no basic or ultrabasic lithology has been reported in the immediate study area. Therefore, Source 2 is interpreted as a geogenic source associated with weathering of the local granitic parent material and secondary weathering products. In such highly evolved granites, Cr, V, Ni, and Co can be hosted in micaceous minerals (e.g., lepidolite, muscovite, and biotite), accessory phases (e.g., tourmaline, zircon, apatite, and Fe–Ti oxides), and secondary clay minerals and Fe–Mn oxides. Weathering can release these elements from primary minerals and redistribute them into fine-grained secondary phases. This interpretation is supported by several lines of evidence. First, the enrichment levels of V, Co, Cr, Ni, and Cu were only mild to moderate relative to the local background (Table 1), suggesting a predominantly geogenic control. Second, Cr, V, Ni, and Co were strongly positively correlated with one another (r = 0.94–0.99, *p* < 0.01; Table 2), indicating a common geogenic source. Third, these elements showed negative trends with bulk density and organic matter (Table 2), consistent with enrichment in finer, clay-rich, and less compacted weathering products. Fourth, the study area is dominated by topaz–lepidolite granite, without basic or ultrabasic rocks. The Cu loading in Source 2 may reflect its association with clay minerals and Fe–Mn oxides, consistent with the positive correlation between Cu and pH.

Source 3 was estimated to explain 30.5% of the potential sources. It was characterized by high loadings of Cd (53.99%), Zn (47.61%), and Mn (47.60%), respectively (Figure 4). The contamination levels of Cd and Mn were both severe (Table 1). Compared to the other heavy metals measured, the *P_i_* for Cd was two orders of magnitude higher. This statistical evidence points toward Source 3 being associated with anthropogenic sources [25,37]. In previous studies, multiple pathways, including mining activities, combustion of coal and waste, application of chemical fertilizers and pesticides, sewage sludge release, industrial waste processing, and atmospheric fallout, lead to Cd’s introduction into the environment [29]. Given that agricultural and industrial activities are relatively limited within the mining area, it is less likely that the severe TDSM Cd contamination is primarily from agricultural or industrial pollution. Instead, it is more likely to originate mainly from mining-related activities within the region. In fact, during the processes of extraction, beneficiation, and smelting, Cd is released into the environment as a by-product [25,37]. Furthermore, mining is also one of the main potential sources of high concentrations of Mn in Chinese soils [13,32]. Thus, Source 3 was tentatively interpreted as a mining-related source, potentially associated with the extraction, beneficiation, and smelting of lepidolite ore. Additionally, the high and relatively uniform Cd concentrations in this lithium tailings pond require a specific explanation. During lepidolite processing, large volumes of Cd-bearing tailings are produced. After hydraulic deposition in the pond, the tailings accumulate in the shallow TDSM layer, resulting in high and relatively uniform Cd concentrations. The mining and smelting source might also explain the relatively low spatial variability of Cd. A mineralogical study at the Dajishan Mine reported that, Cd can substitute for Zn in sphalerite and occur mainly as a trace component within the mineral lattice rather than as discrete Cd minerals [38]. Although that deposit is not a lepidolite deposit, this finding provides a general analogue for the possible structural hosting of Cd in sulfide minerals. Hydraulic transport and settling of fine-grained tailings may also have promoted mixing and contributed to the relatively uniform Cd distribution [39,40], but this remains a hypothesis because direct mineralogical and particle-size evidence is lacking.

## 4. Limitations

Several methodological limitations should be acknowledged. First, at each sampling point, surface TDSM cores from 5 quadrats were composited per depth layer. While compositing reduces analytical costs and provides an average estimate for each point, it also smooths small-scale spatial heterogeneity within the point. As a result, the observed lack of significant vertical differences in heavy metal concentrations may partly reflect the compositing effect rather than genuine environmental homogeneity. Therefore, it should be interpreted with caution because the sampling design (compositing of five quadrats per depth layer) may smooth small-scale heterogeneity. Second, the sampling transect covered only one direction (northwest–southeast) across the tailings pond. Although this direction aligns with the valley axis and the main drainage gradient, a more comprehensive sampling grid (e.g., 2-D grid with additional points across the pond) would be needed to fully characterize the spatial distribution of heavy metals. Future studies should use a nested sampling design to preserve spatial variability [41]. Third, direct mineralogical evidence for Cd hosts in the Yichun tailings is lacking. No XRD, SEM–EDS, EPMA, LA–ICP–MS, or sequential extraction analyses were performed. The proposed Cd homogenization mechanism remains hypothetical and should be verified in future studies using these methods. References [38,39,40] are not site-specific and provide only indirect support. Fourth, our 8-point single-transect design captures the main dispersion gradient but not the full two-dimensional spatial heterogeneity; a denser grid would be needed for robust risk mapping. We recommend that future work include broader sampling and Cd measurements in local crops. Finally, while we have applied rigorous diagnostic procedures (QRobust/QTrue ratio, bootstrap resampling, and displacement analysis) to ensure model stability (EPA PMF 5.0 User Guide), PMF alone cannot definitively prove the physical identity of each source factor. The results estimated by PMF model should be considered as statistical hypotheses that require further verification using independent methods (e.g., Pb isotopic fingerprinting, direct measurement of emission sources).

## 5. Conclusions

The enrichment of surface TDSM heavy metals was found in the Li tailings. Among the measured heavy metals, Cd enrichment was the most severe, while the enrichment levels of other metals were mild or moderate. Within the 0–20 cm layer, heavy metal concentrations in the TDSM showed no significant vertical variation. This likely reflects the mixing and homogenization of tailings by hydraulic deposition and mining-related disturbance, indicating that the surface layer is generally affected by anthropogenic activities. This conclusion is limited to the sampled depth range. The PMF model provided statistical indications that the surface TDSM heavy metals in the Li tailings pond might be associated with mixed anthropogenic source, geogenic source associated with the weathering, and mining-related source, with estimated contribution rates of 25.7%, 43.8%, and 30.5%, respectively.

## Figures and Tables

**Table 1:** Concentrations (mg kg^−1^) of heavy metals in tailing pond and control sites, single-factor enrichment index (*P_i_*) in tailings pond TDSM, and the risk control standard (mg kg^−1^) in China (GB15618-2018). “—” indicates that no risk control standard is specified for this element in GB15618-2018.

	TDSM	Soil in Control Sites	*P_i_*	Risk Control Standard
As	12.95 ± 1.94	11.64 ± 3.63	1.11 ± 0.17	40
Zn	109.81 ± 3.67	73.49 ± 2.17	1.49 ± 0.05	200
Cu	58.35 ± 5.42	21.41 ± 3.58	2.73 ± 0.25	50
Ni	44.25 ± 3.26	20.50 ± 2.21	2.16 ± 0.16	70
Co	21.01 ± 1.25	12.29 ± 1.32	1.71 ± 0.10	—
Mn	809.60 ± 32.33	314.15 ± 22.59	2.58 ± 0.10	—
Cr	88.29 ± 4.27	47.54 ± 4.50	1.86 ± 0.09	150
V	101.97 ± 5.41	91.51 ± 8.72	1.11 ± 0.06	—
Pb	55.79 ± 8.93	29.13 ± 10.09	1.92 ± 0.31	90
Cd	20.16 ± 0.48	0.11 ± 0.01	183.24 ± 4.35	0.3

**Table 2:** Pearson correlation coefficients between TDSM physicochemical properties and heavy metals.

	As	Zn	Cu	Ni	Co	Mn	Cr	V	Pb	Cd
Moisture	0.081	0.082	−0.484	0.38	0.245	−0.27	0.335	0.401	0.036	0.273
Bulk density	−0.504	−0.117	0.636	−0.655	−0.731 *	0.250	−0.743 *	−0.762 *	−0.447	−0.508
pH	−0.004	0.611	0.722 *	−0.166	−0.367	0.814 *	−0.327	−0.368	0.151	−0.672
Organic matter	−0.337	−0.038	0.312	−0.600	−0.672	0.492	−0.663	−0.636	−0.308	−0.361
As		0.625	−0.017	0.061	0.083	0.295	0.212	0.244	0.986 **	0.025
Zn			0.392	0.085	−0.102	0.741 *	−0.02	0.029	0.710 *	−0.236
Cu				−0.458	−0.53	0.733 *	−0.501	−0.538	0.099	−0.524
Ni					0.942 **	−0.336	0.949 **	0.952 **	0.055	0.555
Co						−0.515	0.961 **	0.965 **	0.036	0.740 *
Mn							−0.412	−0.411	0.419	−0.605
Cr								0.990 **	0.182	0.593
V									0.203	0.666
Pb										−0.096

*n* = 8; * denotes *p* < 0.05; ** denotes *p* < 0.01.

## Data Availability

The original contributions presented in this study are included in the article/Supplementary Material. Further inquiries can be directed to the corresponding author.

## Supplementary Materials

The following supporting information can be downloaded at: https://www.mdpi.com/article/10.3390/jox16060188/s1, Table S1: Validation results for heavy metals using values of Certified reference material analysis (CRM); Table S2: Results of Shapiro–Wilk test and Levene’s test.

## References

[1] Xia G., Han C., Chen M., Wang G., Lu K., Zhu J., Yao J. (2025). Risk Assessment of Heavy Metals, Nitrogen, and Phosphorus in Seawater of Taizhou Bay, China. J. Xenobiotics.

[2] Li S., Ni G. (2025). Heavy Metals Environmental Fate in Metallurgical Solid Wastes: Occurrence, Leaching, and Ecological Risk Assessment. J. Xenobiotics.

[3] Qi Q., Hu C.X., Lin J.H., Wang X.H., Tang C.X., Dai Z.M., Xu J.M. (2022). Contamination with multiple heavy metals decreases microbial diversity and favors generalists as the keystones in microbial occurrence networks. Environ. Pollut..

[4] Şenilă M., Levei E.A., Şenilă L.R., Oprea G.M., Roman C.M. (2012). Mercury in soil and perennial plants in a mining-affected urban area from Northwestern Romania. J. Environ. Sci. Health A.

[5] He N., Xie Y. (2025). Responses of soil microbial diversity and composition to heavy metals contamination in the largest lithium mine in China. J. Soil Sci. Plant Nutr..

[6] Zhou X., Zou J., Dai L., Yang X., Xu Z., Liao L., Fan Q., Zhou X. (2025). Characteristics, source analysis, and ecological impacts of soil heavy metals in typical lithium mining areas with recommendations for green mining development: A case study of the Alabo and Guanyinqiao lithium mining areas in Western Sichuan. Geol. China.

[7] Gao J., Yu Y., Wang D., Liu L., Wang W., Hao X., Dai H. (2019). The content and distribution characteristics of heavy metals in root soils in the Jiajika lithium resource area, Western Sichuan Province. Rock Miner. Anal..

[8] Yu F., Wang D., Yu Y., Liu L., Dai H. (2019). Ecological risk assessment of heavy metals in soils of Jiajika lithium deposit. Environ. Sci. Technol..

[9] Zhang W., Long J., Wei Z., Alakangas L. (2016). Vertical distribution and historical loss estimation of heavy metals in an abandoned tailings pond at HTM copper mine, northeastern China. Environ. Earth Sci..

[10] Zhang Y. (2021). Pb-Zn Isotopic Characteristics and Microbial Evolution Mechanisms of Soil-Plant System in Gold/Iron Mining Area. Ph.D. Thesis.

[11] Guo W., Zhao R., Zhang J., Bao Y., Wang H., Yang M., Sun X., Jin F. (2011). Distribution characteristic and assessment of soil heavy metal pollution in the iron mining of Baotou in Inner Mongolia. Environ. Sci..

[12] Ma X., Yang S., Wen J., Wang B., Ren X., Ma L. (2025). Pollution characteristic, ecological and health risk assessment of soil heavy metal in a gold mine tailings pond. J. North. Norm. Univ. (Nat. Sci.).

[13] Chen M., Pan Y., Huang Y., Wang X., Zhang R. (2022). Spatial distribution and sources of heavy metals in soil of a typical lead-zinc mining area. Yangshuo Environ. Sci..

[14] Zhang Y., Li L., Ma S., Deng M., Li X., Wu L., Zhou X. (2023). Pollution evaluation and source analysis of heavy metals in farmland soils around the typical manganese mining Area. Nonferrous Met. (Extract. Metall.).

[15] Liao M., Xie X.M. (2007). Effect of heavy metals on substrate utilization pattern, biomass, and activity of microbial communities in a reclaimed mining wasteland of red soil area. Ecotoxicol. Environ. Saf..

[16] Su J., Wang Z., Liu S., Chen D., Hu Z., Ao Y. (2016). Industrial test of lepidolite flotat ion with BK414as a collector in Yichun tantalum &niobium ore. China Min. Mag..

[17] Zou H. (2026). Li-Ta-rich topaz-lepidolite granite from Yichun (China) exhibits the highest ^87^Sr/^86^Sr ratios for Earth’s rocks: Ages and petrogenesis. J. Asian Earth Sci..

[18] Saleem M., Pierce D., Wang Y., Sens D.A., Somji S., Garrett S.H. (2024). Heavy Metal(oid)s Contamination and Potential Ecological Risk Assessment in Agricultural Soils. J. Xenobiotics.

[19] Peña-Fernández A., Higueras M., Tepanosyan G., Peña Fernández M.Á., Lobo M.C. (2026). Geochemical Patterns and Human Health Risks of Less-Regulated Metal(loid)s in Historical Urban and Industrial Topsoils from Alcalá de Henares, Spain. J. Xenobiotics.

[20] (2017). Soil and Sediment-Determination of Total Metal-Microwave Acid Digestion.

[21] Zhao L., Wang L., Jiang Y., Hu Y., Xu C., Wang L., Li X., Wei L., Guo X., Liu A. (2017). Determination of Elemental Concentrations in Lichens Using ICP-AES/MS. Bio-Protocol.

[22] Liu W., Yuan C., Farooq T.H., Chen P., Yang M., Ouyang Z., Fu Y., Yuan Y., Wang G., Yan W. (2024). Pollution index and distribution characteristics of soil heavy metals among four distinct land use patterns of Taojia River Basin in China. Gondwana Res..

[23] Yuan H., Zhang A., Zhu C., Dang H., Zheng C., Zhang J., Cao C. (2024). Saline Water Irrigation Changed the Stability of Soil Aggregates and Crop Yields in a Winter Wheat–Summer Maize Rotation System. Agronomy.

[24] Wen T., Chen D., Diao K., Yu W., Cai S., Yue K., Huang W., Zheng T. (2025). Spatial distribution, ecological risk, and pollution source analysis of heavy metals in sediments from typical lakes in southern Jiangsu Province. Asian J. Ecotoxicol..

[25] Shang T., Ren Y., Mu Y., Zhang J., Wen C., Zhou J., Zhang Y. (2025). Distribution characteristics and source analysis of heavy metals in sediments from the Tianshui Segment of the Weihe River. Res. Environ. Sci..

[26] Wu X., Ouyang Z., Wang C., Zhou S., Peng Y., Li G., Yang F., Xiang K., Zheng J., Zhao B. (2025). Source analysis and health risk assessment of heavy metal pollution in South Dongting Lake waters. China Environ. Sci..

[27] Chen Y., Weng L., Ma J., Wu X., Li Y. (2019). Review on the last ten years of research on source identification of heavy metal pollution in soils. J. Agro-Environ. Sci..

[28] Lion G.N., Olowoyo J.O. (2013). Population health risk due to dietary intake of toxic heavy metals from Spinacia oleracea harvested from soils collected in and around Tshwane, South Africa. S. Afr. J. Bot..

[29] Li D., Ding Y., Zhang Y., Zhang X., Feng L., Zhang Y. (2024). Heavy metals in a typical industrial area-groundwater system: Spatial distribution, microbial response and ecological risk. Chemosphere.

[30] Zhao X., Huang J., Lu J., Jin L., Sun Y. (2019). Study on the influence of soil microbial community on the long-term heavy metal pollution of different land use types and depth layers in mine. Ecotoxicol. Environ. Saf..

[31] Peng P., Wang W. (2020). Effects of soil heavy metal compounds on the micro-aggregate stability in Kafang tailing pond. Agric. Res. Arid Areas.

[32] Ren Y., Cao W., Xiao S., Li X., Pan D., Wang S. (2024). Research progress on distribution, harm and control technology of heavy metals in soil. Geol. China.

[33] Wei C., Yu S., Zhang Y., Tang Y., Deng K., Liu K. (2025). Vertical distribution characteristics and source apportionment of heavy metals in soils of a coking plant site. Environ. Chem..

[34] Liu Z., Dou X., Mao X., Chen K., Gao H., Hu G. (2025). Ecological risk assessment and source analysis of heavy metals in sediments of Qinghai Lake Basin. Chin. J. Environ. Eng..

[35] Yao Y., Li J., He C., Hu X., Yin L., Zhang Y., Zhang J., Huang H., Yang S., He H. (2021). Distribution characteristics and relevance of heavy metals in soils and colloids around a mining area in Nanjing, China. Bull. Environ. Contam. Toxicol..

[36] Yang J., Yang X., He Z. (2005). Resource and bio-availability of lead in soil. Chin. J. Soil Sci..

[37] Chen X., Lu X., Yang G. (2012). Sources identification of heavy metals in urban topsoil from inside the Xi’an Second Ringroad, NW China using multivariate statistical methods. Catena.

[38] Hu X. (1991). Mineralogical characteristics of sphalerites with high cadmium contents from Dajishan Mine. Acta Geosci. Sin..

[39] Arranz-González J.C., Cala-Rivero V. (2017). Grain sorting effects on geochemical characteristics of sulfide mine tailings: A case study. J. Degraded Min. Lands Manag..

[40] Shao L., Chen Z., Guo X., Tian X., Sun Y., Hong Z., Zhu Y., Jiang W. (2021). Hydraulic classification and sedimentation behaviors of iron tailings. Bull. Eng. Geol. Environ..

[41] Sarker A., Mallick S., Uddin M., Das R.C., Rashid M.H., Alam M.S., Quadir Q.F., Hossen M.Z. (2026). Potential Toxic Elements in Farm Soils and Vegetables of Northern Bangladesh: Impact on Soil Health and Human Safety. J. Xenobiotics.

