# Supplementary material for: Characteristics and Sources of Heavy Metals in Tailings-Derived Surface Material in the Largest Lithium Mine in China by Reserves
Source: J Xenobiot. 2026 Oct 5;16(6):188. doi: 10.3390/jox16060188 (PMC13705763; doi:10.3390/jox16060188)
Supplement: Supplementary file 1 [file jox-16-00188-s001.zip › jox-4540166-supplementary.pdf]

Supplementary Materials: Characteristics and Sources of Heavy Metals in Tailings-Derived Surface Material in the Largest Lithium Mine in China by Reserves

Yajun Xie, Yongqiang Liu, Zixuan Ye and Ning He

Table S1. Validation results for heavy metals using values of Certified reference material analysis (CRM).

	Concentration in CRM	Recovery Rate (%)
As	242 ± 16	104.9
Pb	245 ± 14	97.1
Cu	147 ± 10	107.8
Cd	0.16 ± 0.03	92.7
Zn	172 ± 7	101.2
Ni	38 ± 2	95.8
Co	18 ± 2	106.5
Mn	0.051 ± 0.002	99.6
Cr	113 ± 7	103.4
V	136 ± 7	94.3

Table S2. Results of Shapiro–Wilk test and Levene's test.

	Shapiro-Wilk Test		Levene's Test	
	W	*p*	*F*	*p*
Moisture	0.961	0.301	0.624	0.606
Bulk density	0.977	0.718	1.152	0.345
pH	0.961	0.297	0.630	0.602
Organic matter	0.928	0.034	0.261	0.853
As	0.654	0.000	0.434	0.730
Zn	0.975	0.652	2.954	0.050
Cu	0.81	0.000	0.271	0.846
Ni	0.939	0.069	0.731	0.542
Co	0.946	0.109	0.133	0.940
Mn	0.968	0.44	0.825	0.491
Cr	0.966	0.394	0.154	0.927
V	0.95	0.143	0.023	0.995
Pb	0.736	0.000	0.309	0.819
Cd	0.897	0.005	2.730	0.063
